# Editorial: Autoimmune Thyroid Pathology—Specificity of the Pediatric Age

**DOI:** 10.3389/fendo.2021.645278

**Published:** 2021-02-05

**Authors:** Malgorzata Gabriela Wasniewska, Aneta Monika Gawlik, Tommaso Aversa

**Affiliations:** ^1^ Department of Human Pathology in Adulthood and Childhood, University of Messina, Messina, Italy; ^2^ Department of Pediatrics and Pediatric Endocrinology, Medical University of Silesia, Katowice, Poland

**Keywords:** autoimmune thyroid diseases, Hashimoto autoimmune thyroiditis, Graves’ disease, adolescents, children

Autoimmune thyroid diseases (AITDs) are the most common organ-specific autoimmune disorders and the most frequent cause of acquired thyroid dysfunction in adulthood as well as in childhood. AITDs include two main clinical presentations: Hashimoto’s thyroiditis (HT) and Graves’ disease (GD), both characterized by lymphocytic infiltration of the thyroid parenchyma. Clinical and biochemical features of HT and GD are related to hypothyroidism and thyrotoxicosis, respectively (1). In the pediatric population, the prevalence of AITDs is lower than in adults (1.2–1.3% for HT and 1‰ for GD), but increases in presence of chromosomopathies such as Down and Turner syndromes (2). The most common age of presentation of AITDs is adolescence but they may develop at any time, rarely however in infants. Sexual dimorphism, with a female predominance, is similar to adult population.

Although the pathogenic mechanism of AITDs is still under investigation, the evidence in favor of a genetic basis for AITDs is abundant and the factors that might influence the development of autoimmune disease can be related to genetic susceptibility, chromosomal differences or epigenetics (1).

Aim of this Research Topic was to report the most updated views on epidemiology, pathophysiology, diagnosis, long-term prognosis, treatment and management of AITDs in childhood and adolescence. This Research Topic put together eight original articles, two narrative reviews and one very particular case report, summarizing current knowledge on the pathogenesis, clinical long-term experience, clustering and interrelation with other autoimmune diseases, also on the peculiarity of AITDs in genetic syndromes in pediatric populations.

In the narrative review “Hashimoto Thyroiditis and Dyslipidemia in Childhood”, Vukovic et al. discussed recent findings regarding the effects of AITDs on lipid metabolism and cardiovascular risk (CVR), including the beneficial impact of L-T4 treatment on dyslipidemia and potential usefulness of novel lipid biomarkers, such as proprotein convertase subtilisin/kexin type 9 (PCSK9), non-cholesterol sterols, low-density lipoprotein particle size and number and high-density lipoprotein structure and functionality in pediatric patients with AITDs.

In the second review “Autoimmune Thyroid Disease in Specific Genetic Syndromes in Childhood and Adolescence”, Kyritsi and Kanaka-Gantenbein presented the state of the art on autoimmune and non-autoimmune thyroid pathology in chromosomal and other genetic origin syndromes, in a very detailed and updated way. We want to emphasize the didactic usefulness of this paper for pediatricians and pediatric endocrinologists.

The description, published by Bruns et al., of a rare case of polyautoimmunity including autoimmune thyroiditis, Sjögren’s syndrome, vitiligo and celiac disease, entitled “Unusual presentation of polyautoimmunity and renal tubular acidosis in an adolescent with Hashimoto’s thyroiditis and central pontine myelinolysis”, highlighted the need for pediatricians to be aware of rare accompanying diseases and their complications in “common” pediatric autoimmune diseases like Hashimoto’s thyroiditis and celiac disease.

In the context of this Research Topic, there are also four original retrospective studies that allowed us to increase our clinical knowledge based on the wide experience of the Authors.

Calcaterra et al. analyzed a pediatric population with onset of AITDs to assess gender differences with regard to onset age, disease subtype, pubertal status, autoimmune co-morbidity, family history, and treatment, focusing on the interaction between gender and pubertal stage. In the Authors’ opinion, the gender specific characteristics of disease across puberty might help early diagnosis and clinical management of the thyroid pathology.

Admoni et al., in their study of “Long-Term Follow-Up and Outcomes of Autoimmune Thyroiditis in Childhood,” tried to assess which factors at presentation can predict evolution over time of AITDs in pediatric age.

Kucharska et al. dealt with the topic “Clinical and Biochemical Characteristics of Severe Hypothyroidism Due to Autoimmune Thyroiditis in Children”. Authors concluded that in children with severe hypothyroidism, above all without goiter, the most sensitive clinical symptoms were growth arrest and weight gain. Moreover, the specific biochemical profile closely correlated to the condition involved mostly erythropoiesis, liver function, and kidney function. Furthermore, pituitary enlargement should be considered in every child with severe hypothyroidism.

In a single-center retrospective study, over a 9-year observation period, Glowinska-Olszewska et al. revealed an increasing rate of new diagnosis of type 1 diabetes (T1D) together with a growing overall prevalence of additional autoimmune diseases (AITDs and coeliac) at T1D onset.

Again Glowinska-Olszewska et al. prospectively investigated whether HT could increase CVR in young patients with T1D. The crucial finding of their study was that young patients with T1D and coexisting additional HT had a much more unfavorable profile of classical CVR factors compared to T1D peers without any additional disease. Furtak et al. in their clinical study suggested the protective role of infliximab therapy in the development of AITD and the usefulness of thyroid ultrasound to identify the probable risk for AITD in pediatric patients with Crohn’s disease.

Finally, two very innovative studies on genetic predisposition for autoimmune diseases development have been published on this Research Topic.

Borysewicz-Sanczyk et al. performed a genetic association study of IL2RA, IFIH1, and CTLA-4 polymorphisms with AITD and T1D. Authors confirmed that in children and adolescents the investigated genes loci had a role in T1D, but not in AITD susceptibility.

Furthermore, Sawicka et al. performed an analysis of polymorphisms rs709369-*IL-2RA*, rs7138803-*FAIM2* and rs1748033-*PADI4* in a cohort of adolescents with AITDs followed in two European centers of pediatric endocrinology and demonstrated that these polymorphisms confer a genetic predisposition to develop autoimmune thyroid disorders, especially Graves’ disease.

In conclusion, this Research Topic provides an important and updated contribution on the peculiarity of AITDs in childhood. Several papers highlighted the need for prospective studies to clarify certain pathogenetic, clinical, and also therapeutic aspects of AITDs in childhood.

Finally, AITDs in pediatric age is confirmed as an active and still growing area of research. Increasing knowledge of pathogenetic mechanisms will allow to better determine predisposition to AITD development, to earlier diagnose thyroid gland dysfunction, and to improve treatment.

## Author Contributions

MW, AG, and TA conceptualized, designed, wrote and approved the Editorial. All authors contributed to the article and approved the submitted version.

## Conflict of Interest

The authors declare that the research was conducted in the absence of any commercial or financial relationships that could be construed as a potential conflict of interest.
